# Applying genotyping (TILLING) and phenotyping analyses to elucidate gene function in a chemically induced sorghum mutant population

**DOI:** 10.1186/1471-2229-8-103

**Published:** 2008-10-14

**Authors:** Zhanguo Xin, Ming Li Wang, Noelle A Barkley, Gloria Burow, Cleve Franks, Gary Pederson, John Burke

**Affiliations:** 1 Plant Stress and Germplasm Development Unit, USDA-ARS, 3810 4th Street, Lubbock, TX 79415, USA; 2 PGRCU, USDA-ARS, 1109 Experiment Street, Griffin, GA 30223, USA; 3 Garrison & Townsend Inc, PO Drawer 2420, Hereford, TX 79045, USA

## Abstract

**Background:**

Sorghum [*Sorghum bicolor *(L.) Moench] is ranked as the fifth most important grain crop and serves as a major food staple and fodder resource for much of the world, especially in arid and semi-arid regions. The recent surge in sorghum research is driven by its tolerance to drought/heat stresses and its strong potential as a bioenergy feedstock. Completion of the sorghum genome sequence has opened new avenues for sorghum functional genomics. However, the availability of genetic resources, specifically mutant lines, is limited. Chemical mutagenesis of sorghum germplasm, followed by screening for mutants altered in important agronomic traits, represents a rapid and effective means of addressing this limitation. Induced mutations in novel genes of interest can be efficiently assessed using the technique known as Targeting Induced Local Lesion IN Genomes (TILLING).

**Results:**

A sorghum mutant population consisting of 1,600 lines was generated from the inbred line BTx623 by treatment with the chemical agent ethyl methanesulfonate (EMS). Numerous phenotypes with altered morphological and agronomic traits were observed from M_2 _and M_3 _lines in the field. A subset of 768 mutant lines was analyzed by TILLING using four target genes. A total of five mutations were identified resulting in a calculated mutation density of 1/526 kb. Two of the mutations identified by TILLING and verified by sequencing were detected in the gene encoding caffeic acid *O*-methyltransferase (*COMT*) in two independent mutant lines. The two mutant lines segregated for the expected brown midrib (*bmr*) phenotype, a trait associated with altered lignin content and increased digestibility.

**Conclusion:**

TILLING as a reverse genetic approach has been successfully applied to sorghum. The diversity of the mutant phenotypes observed in the field, and the density of induced mutations calculated from TILLING indicate that this mutant population represents a useful resource for members of the sorghum research community. Moreover, TILLING has been demonstrated to be applicable for sorghum functional genomics by evaluating a small subset of the EMS-induced mutant lines.

## Background

Sorghum (2n = 2x = 20, 7.35 × 10^8 ^bp for 1C nucleus) is a C_4 _crop that displays excellent tolerance to both drought and high temperature stresses [1]. Sorghum has the highest water use efficiency among major crop plants and is unusually tolerant to low soil fertility, traits essential for survival and productivity in arid and semi-arid areas with limited irrigation capability. Worldwide, sorghum is the 5^th ^most important grain crop, providing food and fodder for the inhabitants of drought-susceptible regions . Recently, sorghum has been demonstrated as a viable bioenergy feedstock [2]. Compared with other bioenergy grain crops, sorghum is particularly advantageous because it can be grown profitably on marginal land and therefore, would not remove more fertile land from existing food and fiber production [3].

As a close evolutionary relative of both rice and maize, sorghum research contributes directly to a better understanding of the structure, function, and evolution of cereal genomes [4,5]. Being unusually tolerant to drought and high temperature stresses, sorghum also serves as a repository of genes that have the potential to improve stress tolerance in other crops. Recent progress in sorghum genomic studies has generated a series of important tools that can be used to identify favorable genes or alleles for further enhancement of resistance to abiotic stresses and for improved biofuels-related traits. For example, well-established genetic, physical, and cytological maps facilitate the mapping and identification of genes responsible for important agronomic traits [6-8]. Syntenic alignment of the sorghum genome with those of maize, rice, and other cereals provides important insight into genome evolution [9-11]. Furthermore, the construction of cDNA microarrays provides a platform for high throughput gene discovery [12,13]. An important milestone was the recent completion and posting of the genomic sequence of an inbred sorghum line, BTx623 . Utilization of these important genomic, genetic, and biotechnological resources such as Targeting Induced Local Lesions In Genomes (TILLING) will undoubtedly speed-up the elucidation of sorghum gene function and the identification of candidate genes for improving sorghum germplasm.

TILLING was first developed in *Arabidopsis thaliana *[14] and has been successfully applied to identify knockout mutations and provide allelic mutations in target genes from pathogenic bacteria [15], animals, and plants [16,17]. The application of TILLING to animal species includes *Caenorhabditis elegans *[18], zebrafish [19], and *Drosophila *[20]. The application of TILLING to plant species includes *Arabidopsis *[21], barley [22,23], lotus [24,25], wheat [26], maize [27], *Populus *[28], rice [29,30], pea [31], and soybean [32]. The technique has proven to be valuable in characterizing the function of target genes [18,24,29,30,32,33].

The challenge for researchers is to decipher the function of sorghum genes, particularly those that are unique to the species. Unfortunately, many of the reverse genetic tools, such as T-DNA tagging and transposon-tagging are still not available in sorghum. Although active transposon elements have been identified in sorghum, no viable transposon-tagged population has been established [34,35]. RNAi has emerged as an effective gene knockout/knockdown tool for many but, has yet to be applied to sorghum. Development of RNAi technology requires genetic transformation and very little work on transformation protocols have been undertaken in sorghum due to the concern that transgenes may transmit to wild related species.

In contrast to transgenic methods, chemical mutagenesis can be applied to most species including sorghum. Chemical mutagenesis does not require gene transfer and is therefore not subject to biosafety and extensive regulatory concerns [36]. Chemical mutagenesis has been used in sorghum breeding previously; unfortunately, these mutagenized populations were not annotated and preserved [37,38]. Here, we report the generation of an ethyl methanesulfonate (EMS)-mutagenized sorghum mutant population, phenotyping of mutant lines in the population, TILLING analysis of a subset of mutant lines using four target genes, and verification of the observed mutations with the resultant phenotypes.

## Results

### Generation of mutant population

**Figure 1:**
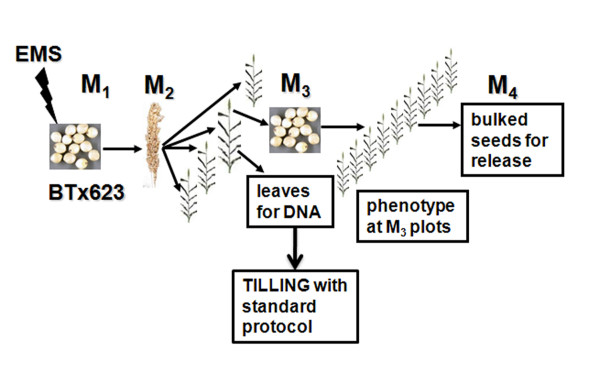
**Mutant population generation**. Dry seeds of sorghum inbred BTx623 are mutagenized with chemical mutagen EMS and germinated to produce M_1 _plants. M_1 _plants are self-fertilized to produce the M_2 _seeds. One fertile M_2 _plant from each M_2 _head row was used to produce M_3 _progenies. Duplicate leaf samples were collected from the same M_2 _plant for extracting DNA used in TILLING analysis. Systematic phenotyping was deferred to M_3 _generation to ensure the mutant phenotypes observed is represented in the M_3 _seed pool. Ten heads for each M_3 _head row were pooled as M_4 _seed pools for public release. Once the DNA is extracted from the mutant population, the DNA is normalized and pooled together as eight-fold pools. The targeted gene is amplified using a forward primer with 700 nm dye label and a reverse primer with an 800 nm dye label attached to the 5' ends. The PCR products are analyzed on LI-COR DNA Analyzer with 700 and 800 nm duel channel following standard TILLING protocol [59].

Chemical mutagen, EMS, was used to generate the mutagenized population because of its high rate of success in many plant species [21]. Previously published reports demonstrate that it is a beneficial strategy to try a range of concentrations of the chemical mutagen being applied to evaluate the toxicity and sterility on germinal tissue before preparing large mutant populations [39]. Therefore, the first step was to test the effect of EMS on sorghum germination. A range of concentrations from 0.1 to 0.6% (v/v) EMS was applied and evaluated. The M_1 _plants were scored for germination, reduced plant height, frequency of leaf chimeras, and fertility of M_1 _panicles. No significant reduction in the germination rate was observed in any of the applied concentrations of EMS from 0.1% to 0.6%. However, seedling growth was severely stunted at concentrations of 0.3% or higher. Even at 0.1%, most M_1 _plants exhibited wrinkled leaves. Over 10% of the M_1 _plants treated with 0.1% EMS had at least one leaf with chimeric white stripes. Based on this result, 0.1%, 0.15%, 0.2%, 0.25%, and 0.3% EMS were used to generate the mutagenized sorghum populations following the scheme outlined in Figure 1.

**Figure 2:**
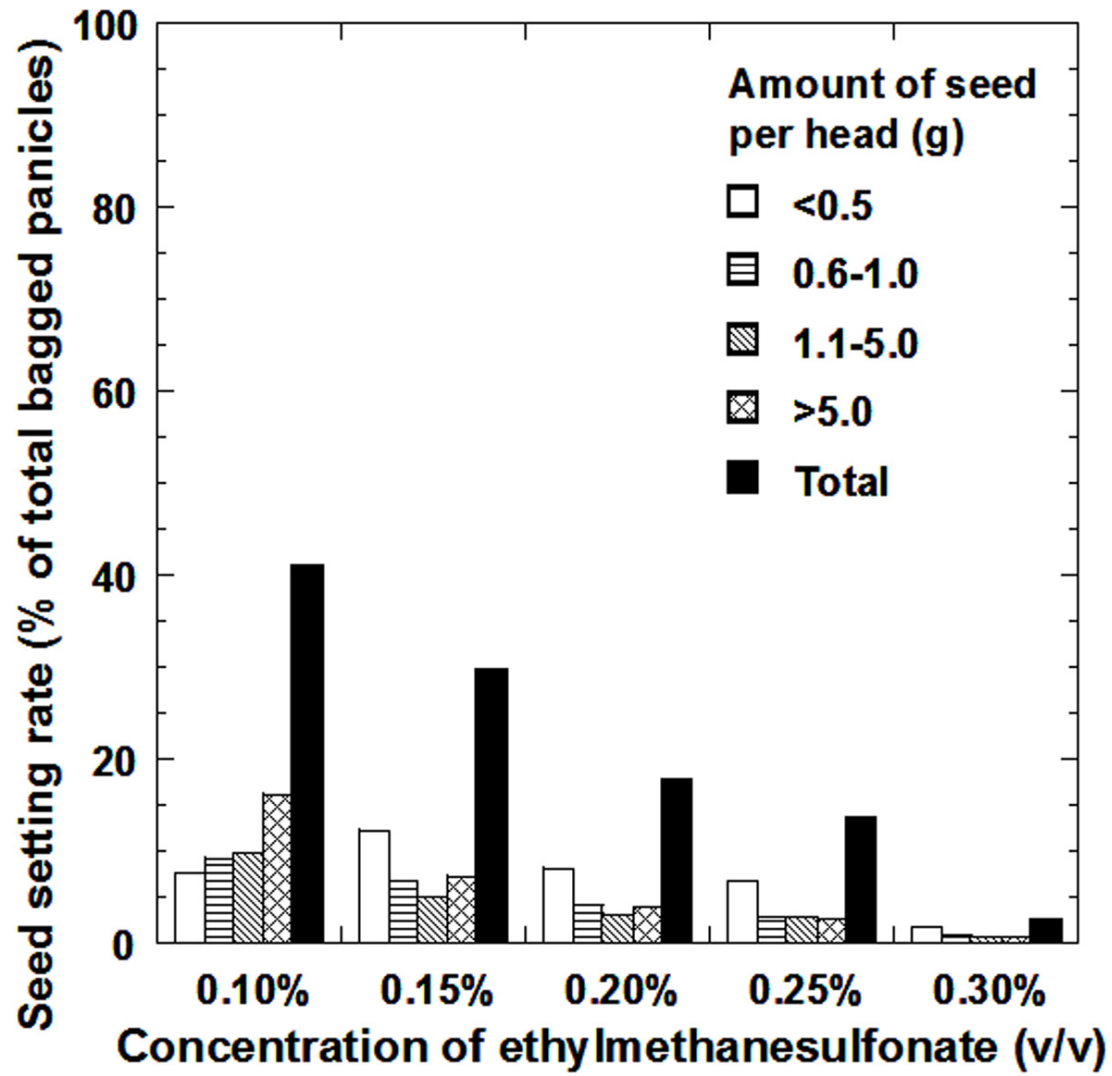
**Histogram detailing the seed setting of EMS-treated M_1 _plants**. Sorghum panicles were harvested manually and threshed individually. The amount of seed set on each panicle was weighed and classified as < 0.5 g (< 20 seeds), 0.6 to 1.0 g (20 to 50 seeds), 1.1 to 5 g (50 to 200 seeds), and > 5.0 g (over 200 seeds). The wild-type BTx623 set ~1200 seeds/panicle on average. The x-axis showed the EMS concentrations used and the panicle classes within each concentration. The y-axis showed the percentage of each panicle class over the total panicles bagged for the concentration of EMS used.

In 2005, about ~50,000 sorghum seeds were treated with EMS (~10,000 for each concentration mentioned above) and field planted at 120,000 seeds per hectare. In a previous study, an examination of resulting M_2 _plants using four hyperpolymorphic sorghum simple sequence repeat (SSR) markers, Xtxp287, Xtxp270, Xtxp51, and Xtxp295 (publicly available, [6]) showed that over 30% of the M_2 _plants were the result of cross pollination from unknown sources (data not shown). In order to prevent cross fertilization with other sorghum varieties growing in the region, each healthy panicle was bagged before anthesis. There was an inverse correlation between M_1 _plant fertility and mutagen dosage (Figure 2). Typically, lowering the treated dosage will decrease the overall mutation rate. Therefore, the key for determining the optimal mutagen dosage is to maximize mutational density while minimizing lethality and aneuploidy [21,40]. Seed setting was greatly affected by EMS treatment. Untreated BTx623 plants set about 40 g (~1,200 seeds) per panicle. After EMS treatment, not only the number of seeds per panicle was greatly reduced but the number of sorghum panicles that set seeds at all also decreased with increasing concentrations of EMS treatment. At a 0.1% concentration of EMS, about 41% of the bagged panicles set seeds (Figure 2). On the other hand, an applied concentration of 0.3% EMS produced only a few panicles that set seeds (with most of them producing fewer than 20 seeds). Moreover, the seeds from the 0.3% treated M_1 _plants had poor germination rates and very few lines produced healthy plants that set seeds in the next generation. Overall, a concentration of 0.25% EMS was the highest dosage at which treated seeds developed into healthy M_1 _plants (13.5%) and produced viable M_2_seeds.

Since the EMS concentrations used to generate this mutagenized sorghum population was relatively low compared with that used in other plants [21,30,32,41], only the M_2 _seeds from 0.25% EMS treated plants were used to conduct the pilot TILLING study. To maximize the utility of the limited M_2 _seeds, 50 (or all if less than 50) of the M_2 _seeds from each M_1 _panicle were planted as a head row. Three panicles from each row were bagged before anthesis. To prevent redundancy of mutations, only one fertile plant from each M_2 _head row were selected to produce M_3 _seeds. The plants were barcoded and leaf tissues were sampled for DNA preparation. A total of 1,246 M_3 _lines were produced in the field. An additional 500 M_3 _lines were produced in the greenhouse following a similar procedure as noted in Figure 1.

### Phenotyping of the mutagenized population

Among the 1,246 M_3 _families planted in the field, 1,160 families had good stand (> 10 healthy plants). Throughout the growing season these families were repeatedly evaluated for phenotypes distinctive from wild-type BTx623. The phenotypes were organized into seedling phenotypes, tillering types, leaf necrosis, senescence, panicle shapes, and seed size (Table 1). In the field, all M_3 _families were inspected closely for novel morphological traits compared to the wild type. Every M_3 _family segregated for at least one distinctive mutant phenotype and many lines segregated for multiple mutant phenotypes at an approximately 1:3 ratio (mutant phenotype to wild type). For example, one line (P5A3) was homozygous for *brown midrib *(*bmr*) but, heterozygous for male sterility and dwarfing. Many lines that segregate for multiple phenotypes can be found online at .

**Table 1:** Frequency of M_3 _families segregating for typical mutations observed in the field

**Phenotype description**	Symbol*	**Number of mutant observed**	**Frequency**
dwarf and semi-dwarf	*dw*	201	17.3%
albino	*w*	171	14.8%
narrow leaf	*nrl*	87	7.5%
multiple tillers	*mtl*	86	7.4%
tiny plants	*tny*	85	7.3%
spot leaf lesion	*sp*	76	6.5%
leaf rolling	*rl*	58	5.0%
erect leaf	*erl*	53	4.6%
short leaf	*sl*	51	4.4%
necrotic leaf	*ncl*	40	3.4%
chlorotic leaf	*chl*	40	3.4%
stacked leaf	*stl*	36	3.1%
adherent leaf	*adl*	35	3.0%
wide leaf	*wdl*	32	2.8%
yellow splotch leaf	*ysp*	25	2.2%
undeveloped panicles	*udp*	22	1.9%
brown midrib	*bmr*	21	1.8%
leaf bronzing	*lbr*	21	1.8%
light green leaf	*lgl*	19	1.6%
twisted leaf	*twl*	17	1.5%
pineapple leaf	*pnl*	14	1.2%
single stalked	*Tx*	13	1.1%
early maturity	*ma*	12	1.0%
zebra crossbands	*zb*	10	0.9%
bloomless	*bm*	10	0.9%
grass leaf	*grl*	8	0.7%
variegated leaf	*var*	7	0.6%
lodging	*ldg*	7	0.6%
leaf striations	*str*	6	0.5%
late flowering	*Ma*	5	0.4%
poor panicle exertion	*exr*	5	0.4%

*Phenotypes were named according to Rooney [60] wherever possible; new phenotypes are named using a three letter code according to [61].

**Figure 3:**
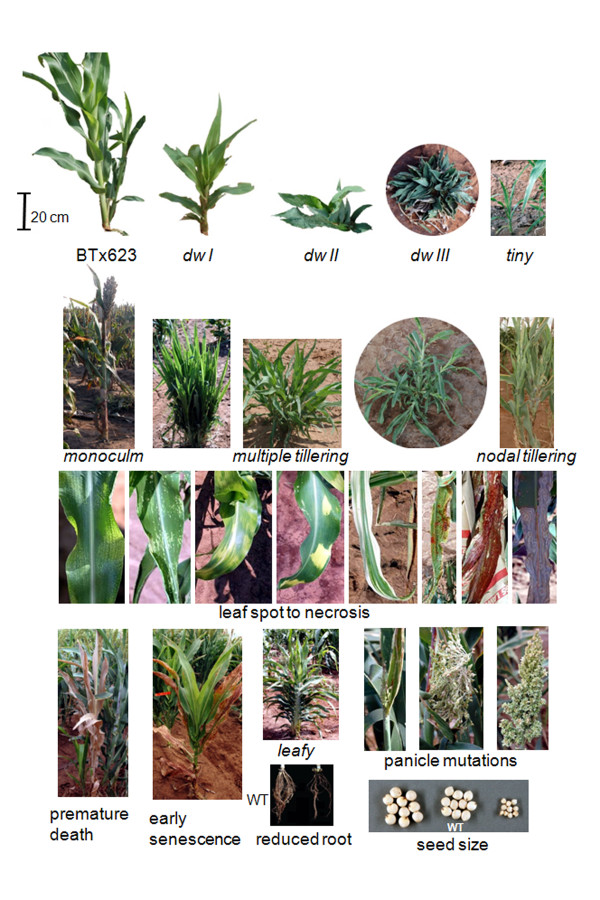
**A gallery of selected mutant phenotypes**. Selected mutant phenotypes were presented to illustrate the diversity mutations observed from this sorghum mutagenized population. Additional mutant phenotypes can be found online .

The observed phenotypes are listed in Table 1 arranged in order from the highest to lowest frequency. Albinism, usually used as an indicator for mutation frequency [41], occurred in 14.8% of the head rows in the M_3 _generation. About 17% of the rows segregated for "dwarf" or "semi-dwarf" plants, the most frequently observed phenotype characterized by reduced height but, largely normal leaf size and stem thickness. Over 6% of the rows segregated for "tiny plants", which differed from "dwarf plants" by reduction in the size of all organs, such as small leaves and thin stems (Figure 3). Leaf lesions, ranging from tiny yellow spots to large blotches of necrosis, were frequently observed (Figure 3). In addition to these frequent mutant features, a number of other mutant phenotypes with altered agronomic traits (such as number of tillers, lodging, early senescence, altered seed size, and flowering time) were also observed. A selection of the mutant phenotypes observed in the field is presented in Figure 3. Further information on the phenotypes observed from the forward genetic screen can be found online at . The assortment and frequency of the various mutant phenotypes observed in the population suggested that it will be a useful resource for screening informative sorghum mutants for forward and reverse genetic studies.

### Identification of mutations by TILLING

Eight-fold pools of genomic DNA from leaf tissues of M_2 _plants were used for TILLING. Four gene targets were selected based on their potential contribution to bioenergy, nutrition, and agronomic performance for high throughput TILLING (Table 2). A total of five mutations were detected from 3.24 Mb of DNA analyzed by TILLING in four gene targets (Table 3). The overall mutation density was calculated to be ~1/526 kb for the EMS generated mutant population (Table 3). This was calculated by dividing the total number of mutations revealed by TILLING by the total base pairs screened, which includes the sum of the total length of the four amplicon sizes × the total number of individuals screened (3,424 bp × 768 individuals). Previous studies have reported the difficulty of tracking mutations on the ends of the fragment (~100 bp) [16,21,30,32]; therefore, 200 bp was subtracted off each amplicon.

**Table 2:** A list of gene targets and primer sequences

Gene names	GenBank ID	Primers	Annealing temperature
1-aminocyclopropane-1-carboxylate oxidase (*ACO1*)	AF079588	5'-AATGGTGGTTCCCGTGATCG-3' 5'-GGCTCCTTGGCCTGGAACTT-3'	69
caffeic acid *O*-methyltransferase (*COMT*)	AY217766	5'-GGCATGGCGTTGTGCTGTAG-3' 5'-CGAGCGACGTACGGAGGACT-3'	69
myoinositol kinase 1 (*MIK1*)	AF124045	5'-CACGTGGCGCTTGACGATAC-3' 5'-CTGCCTGCACCCAGTTGAAA-3'	66
phytochrome A (*PHYA*)	AY466072	5'-GCTTGCTGCCAAGGCAATCT-3' 5'-AGCCAGTGGAATCCCCATGA-3'	63

**Table 3:** Summary of the mutation rate and TILLING mutants

Gene names	Mutants screened	Amplicon size (bp)	Identified mutants	Mutation rate* (kb)
1-aminocyclopropane-1-carboxylate oxidase (*ACO1*)	768	900	0	0
caffeic acid *O*-methyltransferase (*COMT*)	768	1013	2	1/312
myoinositol kinase 1 (*MIK1*)	768	1457	2	1/483
phytochrome A (*PHYA*)	768	854	1	1/502
Total	768	4224	5	1/526

*For calculation of the mutation rate, sequences were removed 100 bp from each end due to the base ambiguity.

EMS mutagenesis is reported to typically produce G:C to A:T transition mutations because it alkylates G residues [42] and these alkylated G residues base pair with T instead of the conventional base pairing with C [36]. Three of the mutations uncovered via TILLING were G:C to A:T transitions as expected. The remaining two mutations detected were A:T to G:C transitions which have also been reported as occurring in other TILLING studies; however, generally these mutations occur at a much lower frequency than the predominant G:C to A:T mutation [30,32]. Of the five mutations identified, one of the mutant lines was determined to be homozygous (P5A3) and the other four were heterozygous. Heterozygosity was evaluated by examining the sequence data and by forming a heteroduplex on a single individual sample, applying the mismatch endonuclease *Cel *I, separating products on a denaturing acrylamide gel, and verifying the presence or absence of the predetermined cleaved fragment. All five mutations detected by TILLING were reexamined by morphological observation and re-sequenced to verify the results. The genetic data were consistent with morphological observations. No mutations were detected for one of the targets, 1-aminocyclopropane-1-carboxylate oxidase (*ACO1*).

**Figure 4:**
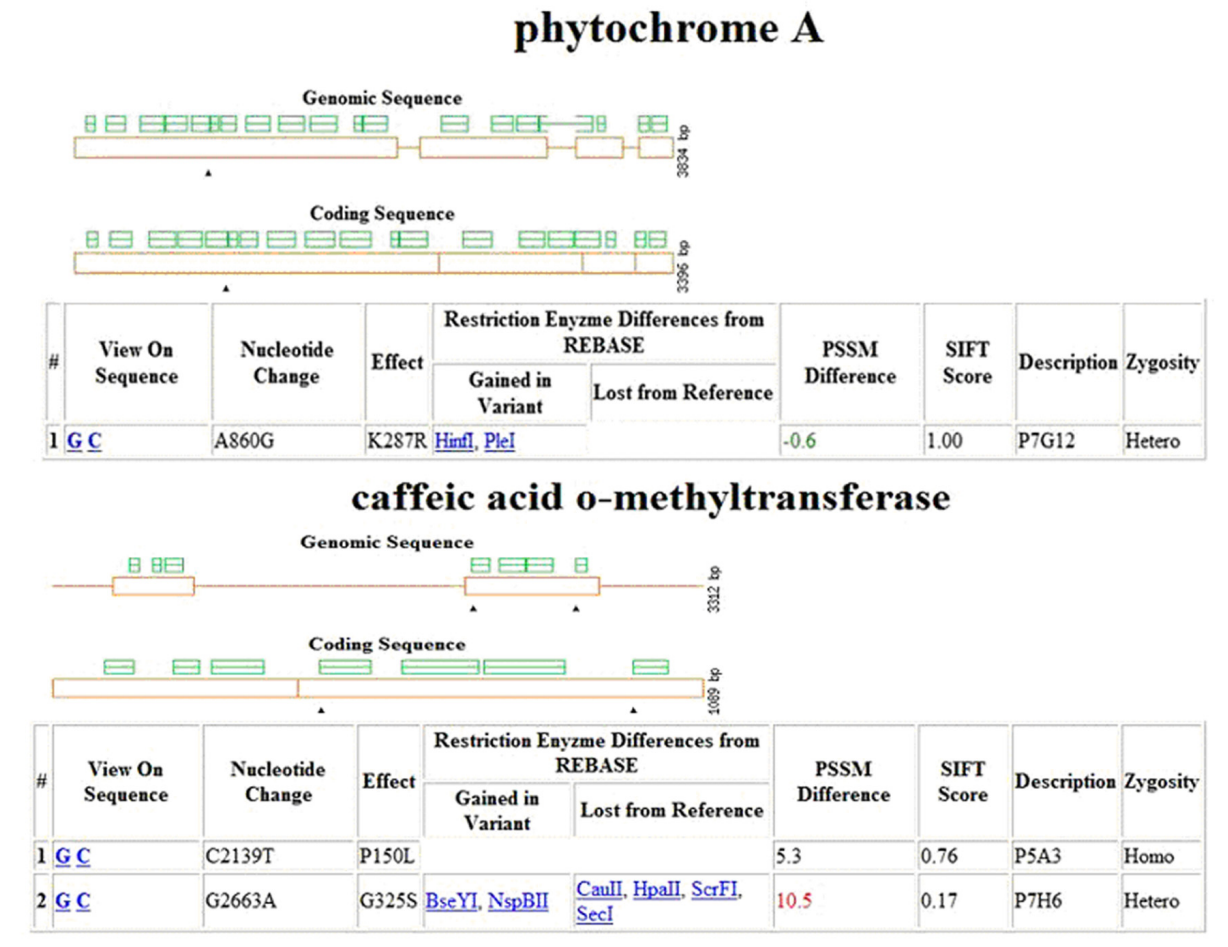
**PARSESNP graphic on *COMT *and *PHYA *mutations revealed by TILLING**. PSSM or SIFT scores shown in red are predicted to be damaging to the protein. PSSM score ≥ 10 indicates a mutation that is more likely to have a damage effect on protein function.

A graph of the target genes marking the location of the induced polymorphisms is shown in Figure 4. A PARSESNP graph was not produced for *ACO1 *or myoinositol kinase 1 (*MIK1*) because no induced mutation was identified in the population or information was not available on the predicted gene structure. The mutations revealed via TILLING from the gene targets caffeic acid *O*-methytransferase (*COMT*) and phytochrome A (*PHYA*) were all determined to be missense mutations. Based on the PSSM difference and SIFT score, the mutation revealed in *COMT *gene for individual P7H6 is predicted to be damaging to the protein. This induced mutation in P7H6 altered the codon from a hydrophobic glycine amino acid to a hydrophilic serine amino acid (Figure 4), which may affect the protein structure. No silent, splice site, or nonsense mutations were identified in this pilot study. However, since there is no information available on the predicted exon/intron borders (gene structure) for *MIK1*, except some similarity to deposited annotated maize genes, the mutations found in this target could represent any of the various mutation classes.

### Verification of identified genotype with observed phenotype

**Figure 5:**
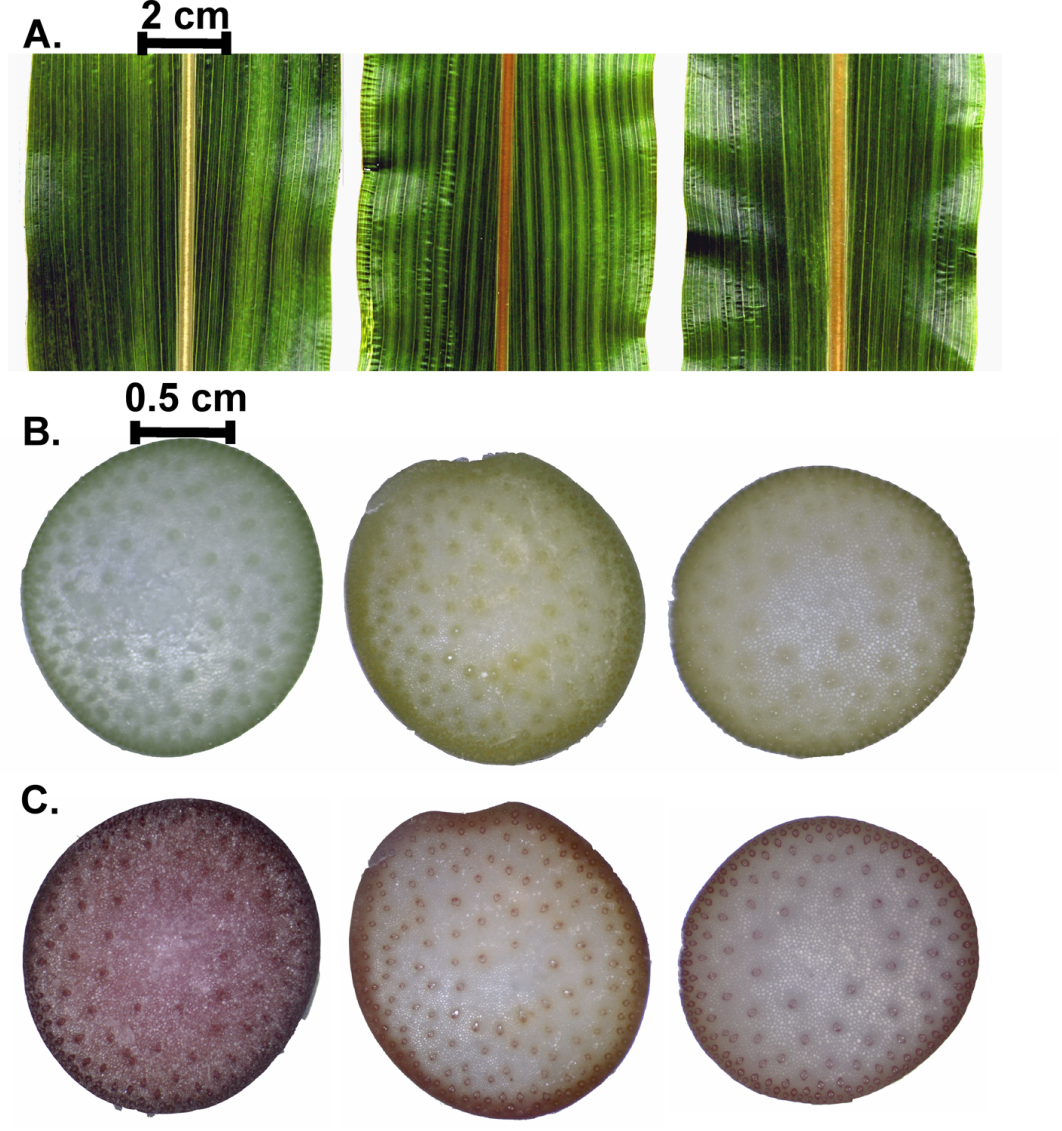
**Phenotype of the *bmr *mutants**. **A**. A section of the leaf blade from BTx623, P5A3, and P7H6. **B**. Cross section of young stems before histochemical staining showing the light brown color in *bmr *mutants. Free-hand sections (~200 μm thickness) were prepared with a razor blade. **C**. The same section as in **B **after histochemical staining for lignin with acidified phloroglucinol showing the reduction in lignin in both mutants. The pictures were arranged as wild-type BTx623, P5A3, and P7H6 from left to right.

Five mutations were identified by TILLING of 768 mutant lines using four target genes. Two mutant lines (P5A3 and P7H6) were identified in the target gene *COMT*, containing missense mutations at different positions in exon 2. Alterations in the *COMT *gene are associated with *bm *or *bmr *mutations in maize and sorghum, respectively, characterized by a brown midrib with reduced lignin content and increased digestibility [43,44]. The two mutant lines (P5A3 and P7H6) identified in this study also showed a similar brown midrib phenotype (Figure 5A) comparable to the previously reported sorghum *bmr *mutants that have nonsense mutations in exon 1 and exon 2 of *COMT*, produced by chemical mutagen diethylsulphonate (Bout and Vermerris, 2003).

Sequencing of individual M_3 _plants from P5A3 and P7H6 indicated that the *COMT *locus in P5A3 mutant was homozygous; whereas the *COMT *locus in P7H6 mutant was heterozygous. In the M_3_generation, the individuals from the mutant line of P7H6 were still segregating for the brown midrib phenotype at an approximately 1:3 ratio, while P5A3 was homozygous for the mutation but segregated for plant height and male sterility. P5A3 and P7H6 for the *COMT *locus are allelic mutants; however, given the morphological traits of P5A3, it may contain additional, unlinked mutations. The mutation in line P5A3 (proline in wild type to leucine in mutant) is predicted by PSSM and SIFT scores to be less damaging to the protein function than the mutation in P7H6 (glycine in wild type to serine in mutant, Figure 4), both lines displayed brown midribs in leaves and light brown color in stem-cross sections (Figure 5A and 5B). Histological staining for lignin (using acidic phloroglucinol) showed that both lines had reduced lignin in stem-cross sections when compared to wild-type BTx623 (Figure 5C). All data to date are consistent with the *bmr *phenotype of the P5A3 and P7H6 lines being a direct result of the mutations within the *COMT *gene.

## Discussion

A TILLING population is developed in sorghum using chemical mutagen, EMS. The success of chemical mutagenesis in plants depends on maximizing both the seed set of the mutagenized plants and the frequency of induced mutations. An adequate mutation frequency is required to keep the number of PCR reactions associated with TILLING to a manageable number. In Till et al. (2007), the authors argued that efficient TILLING requires a population with mutation frequency of ≥ 1 mutation/500 kb to ensure that at least one mutation is found per gel run in the LI-COR DNA analyzer. Achieving this goal requires a high concentration of mutagen or special treatments. For example, treatment with 1.6% EMS produced rice mutant populations that did not meet this threshold level in mutation density [41]. Adequate mutation frequency was, however, achieved through treatment of developing rice zygotes on the panicle immediately after pollination [29].

There are many factors that may affect the induced mutation rate, such as mutagen concentration, length of mutagen application, wash length, treated organs (pollen or seeds), and chosen ecotype (or genotype) of the target species. Seed structure can vary from species to species, which may also influence mutagen efficacy. For example, rice seeds have hulls and the applied chemical needs to penetrate the hulls first and then interact with the embryo to be effective. This may be one reason why rice seeds need a higher dosage for mutagenesis than *Arabidopsis *and sorghum. The concentration of 0.25% EMS was used in our pilot TILLING study as sorghum appears to be very sensitive to EMS treatment. Only about 40% of the M_1 _plants set seeds even at 0.1% EMS concentration (Figure 2). At 0.3%, the commonly used concentration in *Arabidopsis*, very few M_1 _sorghum plants set seeds. Moreover, the seed from fertile M_1_plants had poor germination rates (data not shown). The highest concentration of EMS that produced an acceptable number of fertile plants was 0.25%. At this dose, the population had a mutational density of ~1/526 kb, which is close to the recommended target mutational density (~1/500 kb).

The estimated mutation density was lower than typically reported for *Arabidopsis *(1/300 kb) [21], slightly higher than barley [22] or rice (1/Mb) [41], but similar to the mutation density reported in soybean 1/550 kb [32] and pea 1/669 kb [31]. EMS is reported to have a mutational bias for 5'-PuG-3' sites or a middle G base in a stretch of three or more G bases [45]. Therefore, TILLING gene targets with an elevated G/C content may yield higher mutational densities. The mutational density may change after additional screening of other genes and/or more mutant lines from the population or changing the pooling strategy. An 8-fold pooling strategy was employed in the current study. Lowering the pooling fold (for example, 4-fold pooling) may increase the sensitivity of mutation detection in sorghum. If a suitable mutation density can not be reached with EMS, other chemical mutagens may need to be explored.

Several other factors also impact the establishment of useful TILLING populations in sorghum. Cross pollination must be vigorously controlled to produce a high quality mutagenized population. Under normal growth conditions, sorghum is predominantly self-fertilized with a cross-fertilization rate ranging from 5–10% [46]. After EMS-mutagenesis, cross-fertilization increased dramatically. A previous sorghum mutagenesis attempt was unsuccessful when cloth bags (Lawson Bags, Northfield, IL) failed to prevent cross pollination. In this attempt, cross pollination was prevented by covering the panicles of each mutant generation with rain-proof paper pollination bags (Lawson Bags Northfield, IL) before anthesis. This approach effectively minimized cross pollination. Corn earworm and birds also posed serious threats to the limited seed set in M_1 _plants during the grain-filling period. The paper bag was injected with pesticide to control corn earworm and it was often necessary to put cloth bags over the paper ones to prevent bird damage.

The resulting mutagenized sorghum population has an adequate mutation density and low cross-fertilization, providing a useful community resource for functional analysis of sorghum genes. The variety of visible phenotypes observed in the mutant population is a good indicator of the depth of the genetic lesions, strongly suggesting that the mutant population is altered for multiple traits of agronomic importance. Three allelic mutants (*bmr12*, *bmr18*, and *bmr26*) for the *COMT *locus were previously identified in a sorghum line of P898012 (Bout and Vermerris, 2003). Our two *COMT *mutants (P5A3 and P7H6) in the BTx623 background are phenotypically similar to the previously identified *bmr *mutants, but are the result of missense mutations within different codons. The previous *bmr *mutations have proven to be useful in improving the digestibility of sorghum plants as forage [47]. Since the *bmr *mutations reduce lignin content or improve saccharification of the sorghum stalk, varieties with *bmr *mutations may also serve as improved bioenergy feedstock for cellulosic ethanol production [48-50].

## Conclusion

A mutant population in sorghum has been generated using EMS-mutagenesis. Phenotyping performed in the field, combined with TILLING of four target genes, demonstrated that chemical mutagenesis is an effective approach to generate mutants with altered agronomic traits for genetic studies and to predict the gene function through identification of an allelic series by TILLING. Overall, our results demonstrated that the current sorghum mutant population is an adequate resource for TILLING. As sequence for more target genes becomes available through the nearly completed sorghum sequence project, the gene function for more genes can be elucidated by TILLING of this and additional newly developed mutant populations. Because of the utility of high quality chemical mutagenized populations in sorghum genomic studies, and the inherent difficulties in developing them, it may take a community effort to effectively establish populations with a sufficient number of accessions for TILLING and/or other reverse genetic approaches. Nevertheless, this sorghum mutant population will be a valuable resource to isolate mutants for many other traits. The resource can be accessed for research through contacting the author at zhanguo.xin@ars.usda.gov. Open field day will be held each year for public touring the mutant plots and selection of interested mutants. Scientists who need to select mutants at certain development stage can also be accommodated. Some mutant lines with adequate supply of seeds have already been distributed to a number of sorghum researchers.

## Methods

### Mutagenesis

Sorghum [*Sorghum bicolor *(L.) Moench] inbred line BTx623, which was a parent for several mapping populations in sorghum and the genotype for sequencing the sorghum genome, was used to generate the mutant populations [51-53]. The mutagenesis scheme is outlined in Figure 1. BTx623 seeds were obtained from the National Germplasm Resources of USDA-ARS. Initial observations found that the seedlings from the original seeds showed minor variations in height and panicle size, however, no genetic heterogeneity was detected using 10 publicly available SSR markers. To ensure the homogeneity of the seeds used for mutagenesis, the original line was self-fertilized for six generations by single seed descent (SSD). At every generation, one plant that displayed the most typical characteristics of the original BTx623 was selected for propagating to the subsequent generation. Batches of 100 g of dry seed (~3300 seeds) were soaked with agitation (16 hours at 50 rpm on shaker) in 200 ml of tap water containing EMS concentrations ranging from 0.1 to 0.6% (v/v). The treated seeds were thoroughly washed in about 400 ml of tap water for five hours at ambient temperature, changing the wash water every 30 min. The air-dried seeds were planted at 120,000 seeds per hectare. Before anthesis, each panicle was bagged with a 400 weight rain-proof paper pollination bag (Lawson Bags, Northfield, IL) to prevent cross pollination. After bagging, each bag was injected with 5 ml chlorpyrifos (Dow AgroSciences) at 0.5 ml/liter to control corn earworms that could hatch within the bag and destroy the seeds. Sorghum panicles were harvested manually and threshed individually. Each fertile panicle was planted as an M_2 _head row. Three panicles were bagged for each row before anthesis and only one fertile plant was used to produce the M_3 _seeds. Duplicate leaf samples were collected from the same fertile plant for extracting DNA, and both the leaf samples and the panicle were barcoded. To avoid cross-contamination of leaf samples with dead pollen that could fall onto the leaves during pollen shedding, leaves were thoroughly rinsed with de-ionized water before sampling. The seeds from the barcoded plants were harvested and used to propagate the M_3 _generations. In some cases, because a substantial number of lines could not produce sufficient seeds even at the M_3 _generation, 10 panicles were bagged for each M_3 _head row and pooled as M_4 _seeds. The M_4 _seeds will be distributed to the sorghum research community for forward and/or reverse genetic studies.

### DNA extraction and quantification

Total DNA was extracted with a modified CTAB method [54] and purified with a Qiagen Magattract Plant Kit (Qiagen, Valencia, CA) using lyophilized leaf tissue from individual M_2 _plants. Collecting DNA from only one M_2 _individual derived from each M_1 _plant minimizes sampling identical mutations [30]. Since DNA samples will be proportionally pooled for PCR, the DNA concentration of each sample needed to be precisely quantified. The concentration of DNA samples was first quantified using a Tecan Infinite M200 plate reader (Durham, NC). Subsequently, all DNA samples were loaded on agarose gels and compared with a quantitative DNA ladder (Invitrogen, Carlsbad, CA.). Samples were normalized for PCR and loaded on an agarose gel to ensure dilutions were accurate. Once all samples were verified to be diluted to an equivalent concentration, they were pooled together in eight-fold pools.

### PCR, TILLING, and fragment separation

All PCR reactions were performed in a GeneAmp 9700 (Applied Biosystems^®^, Foster City, CA.) using 96-well microtiter plates. Primer sets were designed for this study from sorghum sequences deposited in GenBank (Table 2). The web based programs Coddle  and Primer3  were used to design the primer sets for this study [55]. The PCR reactions were performed in a 20 μl volume consisting of dH_2_O, 1× PCR buffer (Promega Corp, Madison, WI), 1.5 mM-3 mM MgCl_2 _(Promega Corp.), 0.2 mM dNTPs (Promega Corp.), 0.0625 U *Taq *polymerase (Promega Corp), 0.2 μM unlabeled forward and reverse primers (Operon Biotechnologies, Inc., Huntsville, AL), 0.0125 μM 700 nm and 800 nm 5' labeled (MWG Biotech AG, Germany) forward and reverse primers respectively, 3.125 mg/ml PVP, 0.125 mg/ml BSA (New England BioLabs, Ipswich, MA), and 0.875–1.25 ng DNA. The thermocycling conditions were 95°C for five minutes for initial denaturing, followed by 40 cycles of 95°C for one minute, 63–69°C for one minute, 72°C for one minute, one cycle of 72°C for ten minutes and 4°C hold for storage.

PCR products (~0.2 μl) were separated on a 25 cm KB^Plus ^(LI-COR, Lincoln, NE) polyacrylamide gel (0.25 mm thick) connected to a LI-COR 4300 DNA Analyzer (LI-COR, Lincoln, NE) and quantified on a 3% agarose gel stained with ethidium bromide, along with a low mass ladder (Invitrogen; Carlsbad, CA). The products were separated on a denaturing acrylamide gel to ensure that only a single product was produced; whereas the agarose gel was used to quantify the product to ensure sufficient digestion by the mismatch detection enzyme *Cel *I. The PCR products were heated and cooled in a thermocycler to form a heteroduplex. This consisted of one cycle at 99°C for 10 minutes to inactivate *Taq *polymerase followed by 70 cycles of 20 seconds, starting at 70°C and decreasing 0.3°C per cycle. Once the heteroduplexes were formed, the products were treated with *Cel *I (Transgenomic, Inc. Omaha, NE) according to the manufacturer's instructions. The products were incubated at 42°C for 20 minutes to digest mismatches in the heteroduplex. After the digestion was completed, a stop solution (Transgenomic, Inc.) was added and the products were filtered through a Millipore MultiScreen filter plate that was packed with hydrated Sephadex G-50 medium beads (Sigma, St. Louis, MO). The products were incubated at 80°C for 20–30 minutes to reduce the volume by about a third. Loading dye was added and the PCR products were denatured and loaded onto a polyacrylamide gel attached to a LI-COR 4300 DNA Analyzer for separation.

A total of 768 mutant lines were assayed for mutation induction in the target genes. Once a mutation was revealed, the eight-fold pool (samples: 1–8) was remixed into eight discrete pools consisting of two individuals each (samples: 1&2, 3&4, 5&6, 7&8, 1&3, 2&4, 5&7, and 6&8). If there is a mutation present, then the two pools containing the mutated sample will have the cleaved heteroduplexes in two separate gel lanes and thus, the individual with the mutation will be clearly revealed. Once the positives were identified, the mutant sample, BTx623, and an individual sample from the positive pool was amplified and prepared for sequencing. Amplicons were sequenced either in house using a SequiTherm EXCEL™ II DNA Sequencing Kit (Epicentre^® ^Biotechnologies, Madison, WI) on the LI-COR 4300 DNA Analyzer, or sent to the University of Georgia core genomics facility to be sequenced on a 16 capillary ABI 3100. Prior to sequencing, samples were treated with 1 μl Exonuclease I (10 U/μl) and 1 μl shrimp alkaline phosphatase (SAP,1 U/μl) (GE Healthcare; Piscataway, NJ) and purified with a Qiagen PCR clean up kit (Valencia, CA) to remove all excess nucleotides, primers, enzymes or other impurities. All samples were sequenced multiple times bidirectionally to verify the induced mutation identified from TILLING.

### Phenotyping

Mutation phenotypes were systematically evaluated in the M_3 _generation. Limited phenotyping was conducted at M_2 _generation. Due to large number of the mutants selected at M_2 _generation had poor seed setting, systematic evaluation of mutant phenotypes was deferred to the M_3_generation. Each M_3 _row was carefully inspected at least three times (before flowering, after flowering, and when the majority of the plants reached physiological maturity) during the growing season. Distinguishable phenotypes were recorded and photographed with a digital camera. The frequency of the phenotypes was also recorded.

### Histochemical analysis of cell walls

Free-hand cross sections of young stems were obtained from wild type and mutant plants grown in the greenhouse. Staining for lignin was performed by immersing the sections in acidified phloroglucinol solution based on procedures from [56]. In this method, lignified cell walls were stained as red to dark purple in color. Sections were examined and photographed before and after staining using a Leica MZ6 digital stereomicroscope (Meyer Instrument, Houston, TX).

### Data analysis

The software program Gel Buddy  was used to analyze TILLING gel images and to track the cleaved fragments/variant pools [57]. Once mutants were identified, individual samples were prepared for sequencing. Sequences performed in house were scored with the program E-Seq version 3.0 (LI-COR, Lincoln, NE) and further checked manually for errors. All bidirectional reads were aligned and edited with AlignIR version 2.0 (LI-COR; Lincoln, NE). The web based program PARSESNP [58] was used to produce the graphic showing exons and introns in the gene target and the type of induced mutation uncovered by TILLING.

## Authors' contributions

ZX planned and headed the development of the mutant populations. MLW oversaw the TILLING analysis. NAB conducted the DNA pooling and TILLING experiments. GB conducted histochemical analysis of cell walls. CF conducted field planting and phenotype evaluation. GP and JB co-directed the TILLING and the development of the mutant population. ZX and MLW were primarily responsible for drafting and revising the manuscript with contributions from co-authors. All authors read and approved the final manuscript.

## **Disclaimer**

Mention of trade names or commercial products in this article is solely for the purpose of providing specific information and does not imply recommendation or endorsement by the U.S. Department of Agriculture.

## References

[B1] Doggett H (1988). Sorghum.

[B2] Wang D, Bean S, McLaren J, Seib P, Madl R, Tuinstra M, Shi Y, Lenz M, Wu X, Zhao R (2008). Grain sorghum is a viable feedstock for ethanol production. J Ind Microbiol Biotechnol.

[B3] Rooney WL (2004). Sorghum improvement – integrating traditional and new technology to produce improved genotypes. Adv Agron.

[B4] Paterson AH (2008). Genomics of sorghum. Int J Plant Genomics.

[B5] Mullet JE, Klein RR, Klein PE (2002). *Sorghum bicolor *– an important species for comparative grass genomics and a source of beneficial genes for agriculture. Curr Opin Plant Biol.

[B6] Menz MA, Klein RR, Mullet JE, Obert JA, Unruh NC, Klein PE (2002). A high-density genetic map of *Sorghum bicolor *(L.) Moench based on 2926 AFLP, RFLP and SSR markers. Plant Mol Biol.

[B7] Kim JS, Klein PE, Klein RR, Price HJ, Mullet JE, Stelly DM (2005). Molecular cytogenetic maps of sorghum linkage groups 2 and 8. Genetics.

[B8] Klein PE, Klein RR, Vrebalov J, Mullet JE (2003). Sequence-based alignment of sorghum chromosome 3 and rice chromosome 1 reveals extensive conservation of gene order and one major chromosomal rearrangement. Plant J.

[B9] Bennetzen JL, Ramakrishna W (2002). Numerous small rearrangements of gene content, order and orientation differentiate grass genomes. Plant Mol Biol.

[B10] Bowers JE, Arias MA, Asher R, Avise JA, Ball RT, Brewer GA, Buss RW, Chen AH, Edwards TM, Estill JC (2005). Comparative physical mapping links conservation of microsynteny to chromosome structure and recombination in grasses. Proc Natl Acad Sci USA.

[B11] Jaiswal P, Ni J, Yap I, Ware D, Spooner W, Youens-Clark K, Ren L, Liang C, Zhao W, Ratnapu K (2006). Gramene: a bird's eye view of cereal genomes. Nucleic Acids Res.

[B12] Buchanan CD, Lim S, Salzman RA, Kagiampakis I, Morishige DT, Weers BD, Klein RR, Pratt LH, Cordonnier-Pratt MM, Klein PE (2005). *Sorghum bicolor *'s transcriptome response to dehydration, high salinity and ABA. Plant Mol Biol.

[B13] Salzman RA, Brady JA, Finlayson SA, Buchanan CD, Summer EJ, Sun F, Klein PE, Klein RR, Pratt LH, Cordonnier-Pratt MM (2005). Transcriptional profiling of sorghum induced by methyl jasmonate, salicylic acid, and aminocyclopropane carboxylic acid reveals cooperative regulation and novel gene responses. Plant Physiol.

[B14] McCallum CM, Comai L, Greene EA, Henikoff S (2000). Targeting induced local lesions IN genomes (TILLING) for plant functional genomics. Plant Physiol.

[B15] Kim MJ, Hirono I, Aoki T (2005). Detection of quinolone-resistance genes in *Photobacterium damselae *subsp. *piscicida *strains by targeting-induced local lesions in genomes. J Fish Dis.

[B16] Barkley N, Wang M (2008). Application of TILLING and EcoTILLING as reverse genetic approaches to elucidate the function of genes in plants and animals. Curr Genomics.

[B17] Till BJ, Zerr T, Comai L, Henikoff S (2006). A protocol for TILLING and Ecotilling in plants and animals. Nat Protoc.

[B18] Gilchrist EJ, O'Neil NJ, Rose AM, Zetka MC, Haughn GW (2006). TILLING is an effective reverse genetics technique for *Caenorhabditis elegans*. BMC Genomics.

[B19] Wienholds E, van Eeden F, Kosters M, Mudde J, Plasterk RH, Cuppen E (2003). Efficient target-selected mutagenesis in zebrafish. Genome Res.

[B20] Winkler S, Schwabedissen A, Backasch D, Bokel C, Seidel C, Bonisch S, Furthauer M, Kuhrs A, Cobreros L, Brand M (2005). Target-selected mutant screen by TILLING in *Drosophila*. Genome Res.

[B21] Greene EA, Codomo CA, Taylor NE, Henikoff JG, Till BJ, Reynolds SH, Enns LC, Burtner C, Johnson JE, Odden AR (2003). Spectrum of chemically induced mutations from a large-scale reverse-genetic screen in *Arabidopsis*. Genetics.

[B22] Caldwell DG, McCallum N, Shaw P, Muehlbauer GJ, Marshall DF, Waugh R (2004). A structured mutant population for forward and reverse genetics in barley (*Hordeum vulgare *L.). Plant J.

[B23] Talame V, Bovina R, Sanguineti MC, Tuberosa R, Lundqvist U, Salvi S (2008). TILLMore, a resource for the discovery of chemically induced mutants in barley. Plant Biotechnol J.

[B24] Horst I, Welham T, Kelly S, Kaneko T, Sato S, Tabata S, Parniske M, Wang TL (2007). TILLING mutants of *Lotus japonicus *reveal that nitrogen assimilation and fixation can occur in the absence of nodule-enhanced sucrose synthase. Plant Physiol.

[B25] Perry JA, Wang TL, Welham TJ, Gardner S, Pike JM, Yoshida S, Parniske M (2003). A TILLING reverse genetics tool and a web-accessible collection of mutants of the legume *Lotus japonicus*. Plant Physiol.

[B26] Slade AJ, Fuerstenberg SI, Loeffler D, Steine MN, Facciotti D (2005). A reverse genetic, nontransgenic approach to wheat crop improvement by TILLING. Nat Biotechnol.

[B27] Till BJ, Reynolds SH, Weil C, Springer N, Burtner C, Young K, Bowers E, Codomo CA, Enns LC, Odden AR (2004). Discovery of induced point mutations in maize genes by TILLING. BMC Plant Biol.

[B28] Gilchrist EJ, Haughn GW, Ying CC, Otto SP, Zhuang J, Cheung D, Hamberger B, Aboutorabi F, Kalynyak T, Johnson L (2006). Use of Ecotilling as an efficient SNP discovery tool to survey genetic variation in wild populations of *Populus trichocarpa*. Mol Ecol.

[B29] Suzuki T, Eiguchi M, Kumamaru T, Satoh H, Matsusaka H, Moriguchi K, Nagato Y, Kurata N (2008). MNU-induced mutant pools and high performance TILLING enable finding of any gene mutation in rice. Mol Genet Genomics.

[B30] Till BJ, Cooper J, Tai TH, Colowit P, Greene EA, Henikoff S, Comai L (2007). Discovery of chemically induced mutations in rice by TILLING. BMC Plant Biol.

[B31] Triques K, Sturbois B, Gallais S, Dalmais M, Chauvin S, Clepet C, Aubourg S, Rameau C, Caboche M, Bendahmane A (2007). Characterization of *Arabidopsis thaliana *mismatch specific endonucleases: application to mutation discovery by TILLING in pea. Plant J.

[B32] Cooper JL, Till BJ, Laport RG, Darlow MC, Kleffner JM, Jamai A, El-Mellouki T, Liu S, Ritchie R, Nielsen N (2008). TILLING to detect induced mutations in soybean. BMC Plant Biol.

[B33] Gilchrist EJ, Haughn GW (2005). TILLING without a plough: a new method with applications for reverse genetics. Curr Opin Plant Biol.

[B34] Carvalho CH, Boddu J, Zehr UB, Axtell JD, Pedersen JF, Chopra S (2005). Genietic and molecular characterization of *Candystripel *transposition events in sorghum. Genetica.

[B35] Chopra S, Brendel V, Zhang J, Axtell JD, Peterson T (1999). Molecular characterization of a mutable pigmentation phenotype and isolation of the first active transposable element from *Sorghum bicolor*. Proc Natl Acad Sci USA.

[B36] Henikoff S, Till BJ, Comai L (2004). TILLING. Traditional mutagenesis meets functional genomics. Plant Physiol.

[B37] Sree-Ramulu K (1970). Sensitivity and induction of mutations in sorghum. Mutation Res.

[B38] Jenks MA, Joly RJ, Peters PJ, Rich PJ, Axtell JD, Ashworth EN (1994). Chemically induced cuticle mutation affecting epidermal conductance to water vapor and disease susceptibility in *Sorghum bicolor *(L.) Moench. Plant Physiol.

[B39] Till BJ, Reynolds SH, Greene EA, Codomo CA, Enns LC, Johnson JE, Burtner C, Odden AR, Young K, Taylor NE (2003). Large-scale discovery of induced point mutations with high-throughput TILLING. Genome Res.

[B40] Weil CF, Monde R-A (2007). Getting the Point – Mutations in Maize. Crop Sci.

[B41] Wu JL, Wu C, Lei C, Baraoidan M, Bordeos A, Madamba MR, Ramos-Pamplona M, Mauleon R, Portugal A, Ulat VJ (2005). Chemical- and irradiation-induced mutants of indica rice IR64 for forward and reverse genetics. Plant Mol Biol.

[B42] Comai L, Henikoff S (2006). TILLING: practical single-nucleotide mutation discovery. Plant J.

[B43] Bout S, Vermerris W (2003). A candidate-gene approach to clone the sorghum *Brown midrib *gene encoding caffeic acid *O*-methyltransferase. Mol Genet Genomics.

[B44] Vignols F, Rigau J, Torres MA, Capellades M, Puigdomenech P (1995). The *brown midrib3 *(*bm3*) mutation in maize occurs in the gene encoding caffeic acid *O*-methyltransferase. Plant Cell.

[B45] Bentley A, MacLennan B, Calvo J, Dearolf CR (2000). Targeted recovery of mutations in *Drosophila*. Genetics.

[B46] Ellstrand NC, Foster KW (1983). Impact of population structure on the apparent outcrossing rate of grain sorghum (*Sorghum bicolor*). Theor Appl Genet.

[B47] Aydin G, Grant RJ, O'Rear J (1999). Brown midrib sorghum in diets for lactating dairy cows. J Dairy Sci.

[B48] Porter KS, Anxtell JD, Lechtenberg VL, Colenbrander VF (1978). Phenotype, fiber composition, and in vitro dry matter disappearance of chemically induced brown midrib (*bmr*) mutants of sorghum. Crop Sci.

[B49] Chang MCY (2007). Harnessing energy from plant biomass. Curr Opin Chem Biol.

[B50] Vermerris W, Saballos A, Ejeta G, Mosier NS, Ladisch MR, Carpita NC (2007). Molecular breeding to enhance ethanol production from corn and sorghum stover. Crop Sci.

[B51] Bhattramakki D, Dong J, Chhabra AK, Hart GE (2000). An integrated SSR and RFLP linkage map of *Sorghum bicolor *(L.) Moench. Genome.

[B52] Subudhi PK, Nguyen HT (2000). Linkage group alignment of sorghum RFLP maps using a RIL mapping population. Genome.

[B53] Xu JC, Weerasuriya YM, Bennetzen JL (2001). Construction of genetic map in sorghum and fine mapping of the germination stimulant production gene response to *Striga asiatica*. Yi Chuan Xue Bao.

[B54] Richards E, Reichardt M, Rogers S, Ausubel MF, Brent R, E KR, Moore DD, Seidman JG, Smith JA, Struhl K (1994). Preparation of genomic DNA from plant tissue. Current protocols in molecular biology.

[B55] Rozen S, Skaletsky H (2000). Primer3 on the WWW for general users and for biologist programmers. Methods Mol Biol.

[B56] Ruzin S (1999). Histochemistry and cytochemistry. Plant microtechnique and microscopy.

[B57] Zerr T, Henikoff S (2005). Automated band mapping in electrophoretic gel images using background information. Nucleic Acids Res.

[B58] Taylor NE, Greene EA (2003). PARSESNP: A tool for the analysis of nucleotide polymorphisms. Nucleic Acids Res.

[B59] Till BJ, Colbert T, Tompa R, Enns LC, Codomo CA, Johnson JE, Reynolds SH, Henikoff JG, Greene EA, Steine MN (2003). High-throughput TILLING for functional genomics. Methods Mol Biol.

[B60] Rooney WL, Smith CW, Frederiksen RA (2000). Genetics and Cytogenetics. Sorghum: Origin, History, Technilogy, and Production.

[B61] Neuffer MG, Coe EH, Wessler SR (1997). Mutants of maize.

